# Prevalence, Genetic Homogeneity, and Antibiotic Resistance of Pathogenic *Yersinia enterocolitica* Strains Isolated from Slaughtered Pigs in Bulgaria

**DOI:** 10.3390/antibiotics12040716

**Published:** 2023-04-06

**Authors:** Maya Angelovska, Maya Margaritova Zaharieva, Lyudmila L. Dimitrova, Tanya Dimova, Irina Gotova, Zoltan Urshev, Yana Ilieva, Mila Dobromirova Kaleva, Tanya Chan Kim, Sevda Naydenska, Zhechko Dimitrov, Hristo Najdenski

**Affiliations:** 1 The Stephan Angeloff Institute of Microbiology, Bulgarian Academy of Sciences, 26 Akad. G. Bonchev Str., 1113 Sofia, Bulgaria; 2 Institute of Biology and Immunology of Reproduction, Bulgarian Academy of Sciences, 1113 Sofia, Bulgaria; 3 LB Bulgaricum Plc., R&D Department, 14 Malashevska Str., 1000 Sofia, Bulgaria; 4 University Multiprofile Hospital for Active Treatment, Alexandrovska, Medical University, 1 Georgi Sofiski Str., 1431 Sofia, Bulgaria

**Keywords:** pathogenic *Yersinia enterocolitica*, pigs, PCR, virulence genes, antibiotic resistance, pulsed-field gel electrophoresis

## Abstract

Yersiniosis is the third most commonly reported foodborne zoonosis in the European Union. Here, we evaluated the prevalence of pathogenic *Yersinia enterocolitica* among healthy pigs (as a major reservoir) in a slaughterhouse in Bulgaria. A total of 790 tonsils and feces from 601 pigs were examined. Isolation and pathogenicity characterization was carried out by the ISO 10273:2003 protocol and Polymerase Chain Reaction (PCR), detecting the *16S rRNA* gene, attachment and invasion *locus* (*ail*), *Yersinia* heat-stable enterotoxin (*ystA*), and *Yersinia* adhesion (*yadA*) genes. Genetic diversity was assessed by pulsed-field gel electrophoresis (PFGE), and antimicrobial resistance by the standard disk diffusion method. Of all the pigs tested, 6.7% were positive for *Y. enterocolitica*. All isolates belonged to *Y. enterocolitica* bioserotype 4/O:3. *ail*, and *ystA* genes were detected in all positive strains (n = 43), while the plasmid *Yersinia* virulence plasmid (pYV) was detected in 41. High homogeneity was observed among the strains, with all strains susceptible to ceftriaxone, amikacin and ciprofloxacin, and resistant to ampicillin. In conclusion, a low prevalence of *Y. enterocolitica* 4/O:3 was found in healthy pigs slaughtered in Bulgaria, not underestimating possible contamination of pork as a potential risk to consumer health.

## 1. Introduction

*Yersinia enterocolitica* is a Gram-negative rod within the genus *Yersinia*. The genus comprises 26 species, only 3 of which are known to be human pathogens [1]. *Yersinia pestis* is the causative agent of plague, while *Y. enterocolitica* and *Yersinia pseudotuberculosis* are significant foodborne pathogens associated with yersiniosis. Yersiniosis is an important zoonotic disease with a wide range of clinical symptoms. Symptoms may vary from mild self-limiting acute gastroenteritis to serious complications, systemic infection, and septicemia [2]. According to the European Food Safety Authority (EFSA), yersiniosis was the third most commonly reported zoonosis in Europe in 2021, an increase of 11.8% as compared to 2020, and a total of 6789 confirmed human cases [3]. Additionally, of the two pathogenic species, *Y. enterocolitica* caused the majority (98.1%) of human infections [3].

*Y. enterocolitica* is characterized by strong heterogeneity within the species. According to their biochemical activity, *Y. enterocolitica* strains are classified into 6 different groups: 1A, 1B, 2, 3, 4, and 5. The biotypes differ in terms of geographic distribution, ecological niches, and pathogenicity. Biotype 1A is widely distributed in the environment and regarded as non-pathogenic to animals and humans. Most of the human pathogenic strains belong to biotypes 1B, 2, 3, 4, and 5, of which 1B is considered highly pathogenic. In addition to biotypes, there are more than 70 serotypes of circulating *Y. enterocolitica*, some of which, such as O:3, O:5,27, O:8, and O:9, are shown to have been frequently associated with infection in humans [4]. *Y. enterocolitica* bioserotype 4/O:3, known as a “pig bio- and -serotype”, is the predominant causative strain of human yersiniosis over other bioserotypes [2,5]. Pigs are considered to be the major reservoir for human pathogenic strains [6]. Bacteria persist in the lymphatic tissue of healthy pigs and are frequently disseminated to porcine carcasses during the slaughter process [7,8,9,10,11]. Although *Y. enterocolitica* can be isolated from carcasses, tongues, and feces, the porcine tonsils are still the most important source for bacterial isolation [9,12].

The pathogenicity of *Y. enterocolitica* is due to different chromosomal- and plasmid-encoded virulence determinants. The most important determinants associated with clinical infection are the attachment and invasion locus (*ail*), *Yersinia* heat-stable enterotoxin (*ystA*), *Yersinia* adhesin (*yadA*) genes, invasin (*invA*)*, Yersinia* outer membrane protein virulon (*yop*), low-calcium response regulon (*Lcr*), mucoid *Yersinia* factor (*myfA*)*, Yersinia enterocolitica* chromosomal modulator (*ymoA*), and *Yersinia* virulence regulon (*virF*) genes [6,13]. Their detection provides reliable information about the risk of emerging infection in humans. However, detection of plasmid genes is always challenging because of the temperature dependence and easy loss of the plasmid during repeated cultivation reviewed by Bhaduri and Smith [14].

Among the molecular methods for genotyping of *Y*. *enterocolitica*, pulsed-field gel electrophoresis (PFGE) is commonly used as the gold standard in epidemiological investigations [6]. Moreover, research on the genetic relationship of the isolated strains is an opportunity to describe porcine and human *Y. enterocolitica* isolates, and/or the presence of cross contamination. Thus, this typing method can explain the circulation of strains and the correlation between them.

Relevant in vitro assays have proved that *Y. enterocolitica* strains isolated from slaughtered pigs are susceptible to many antibiotics, such as tetracycline, aminoglycosides, third generation cephalosporins, fluoroquinolones, and resistant to aminopenicillins and first-generation cephalosporins [7,10,15]. Heterogeneity of the antimicrobial resistance profile is shown to depend on the bioserotype and geographic distribution [16]. Multidrug-resistant *Y. enterocolitica* bioserotype 4/O:3 isolates from pigs have increased lately [17]. Their transmission highlights the importance of monitoring the resistance of circulating strains.

There are no data on the occurrence, virulence potential, genetic relationship, and antimicrobial resistance of *Y. enterocolitica* strains isolated from pigs of slaughter age in Bulgaria. The availability of such data would provide scientific outputs for the reports of EFSA. Thus, the aim of this study was to detect the prevalence of pathogenic *Y. enterocolitica* strains in healthy pigs from a slaughterhouse in Bulgaria and to determine their virulence-associated genes, genetic relationship, and susceptibility to different antibiotics.

## 2. Results

### 2.1. Detection of Yersinia enterocolitica

**Figure 1:**
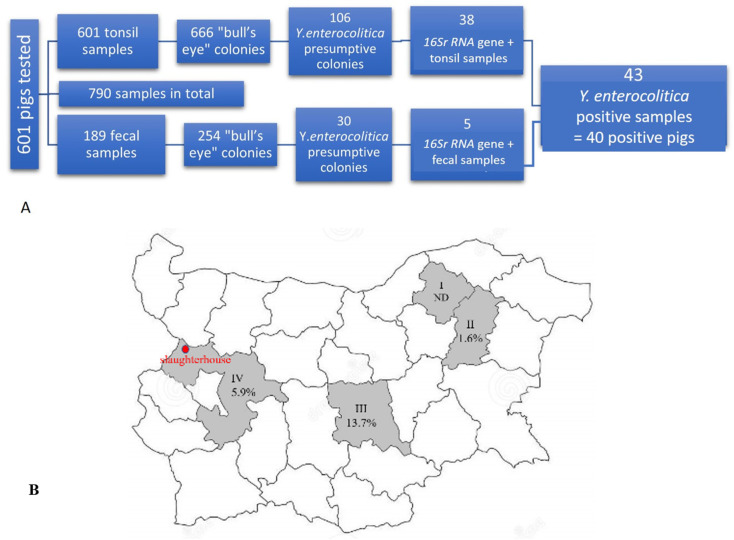
Numbers of pigs and samples tested for *Yersinia enterocolitica* (**A**) and the distribution of the positive pigs per region in Bulgaria (**B**). Regions are presented in roman numerals I: Razgrad region (Farm I), II: Shumen region (Farm II), III: Stara Zagora region (Farm III), and IV: Sofia region (Farms IV, V, VI, and VII). The red dot indicates the location of the slaughterhouse (Kostinbrod city).

An overall of 601 slaughtered pigs were tested for the presence of *Y. enterocolitica* (Figure 1A). Of these, 790 samples were collected, including 601 tonsil samples (tonsil sample from each pig) and 189 fecal samples from 189 pigs (only). From the aforementioned samples (n = 790), 920 colonies were identified on Cefsulodin-Irgasan-Novobiocin (CIN) agar, comprising 666 colonies from tonsil samples and 254 colonies from fecal samples (Figure 1A). Morphological analysis confirmed that all colonies exhibited features of the genus *Yersinia*. After undergoing preliminary biochemical analysis, 136 isolated colonies were identified as presumptive *Y. enterocolitica* based on their degradation of urea, positive catalase test, negative oxidase test, and lack of tryptophan deaminase activity. Subsequently, 43 *Y. enterocolitica* isolates (38 from tonsil samples and 5 from fecal samples) originating from 40 pigs were confirmed using the *16S rRNA* gene (Figure 1A).

Total prevalence of *Y. enterocolitica* in slaughter age pigs was calculated to be 6.7% (40/601 pigs). As shown in Figure 1B, the frequency of *Y. enterocolitica* positive pigs varied according to the region of origin. It was higher among the pigs derived from Stara Zagora region (13.7%, 19/139, Farm III), followed by those from Sofia region (5.9%, 19/324, Farms IV, V, VI, and VII), and the lowest frequency was detected in pigs from Shumen region (1.6%, 2/126, Farm II). There were no positive pigs from Razgrad region (0/12, Farm I). In general, the positive animals originated from six farms (Farms II–VII), located in Stara Zagora, Sofia, and Shumen. All *Y. enterocolitica* strains were isolated only during the cold season (of all sampling periods)—from October to March (2016–2021).

### 2.2. Biotyping and Serotyping

Bioserotyping was performed on the 43 confirmed isolates of *Y. enterocolitica*. All isolates were determined as *Y. enterocolitica* serotype O:3 and biotype 4 based on trehalose utilization, detection of indole, tween esterase and pyrazinamidase activity, and esculin and salicin hydrolysis.

### 2.3. Detection of Virulence Genes

PCR analysis revealed that all *Y. enterocolitica* 4/O:3 isolates (n = 43) were positive for the chromosomally encoded virulence genes *ail* and *ystA*. In 41 isolates, the pYV-coded *yadA* gene was detected by PCR analysis, while 21 were proved positive for pYV by phenotypic assay on Congo red-magnesium oxalate (CR-MOX) agar. The prevalence of *ail-* and *ystA-*positive *Y. enterocolitica* isolates among pigs was calculated to be 6.7% (40/601), while the prevalence of the *yadA* gene was 6.3% (38/601).

### 2.4. Genetic Diversity of Y. enterocolitica Strains Determined by PFGE

**Figure 2:**
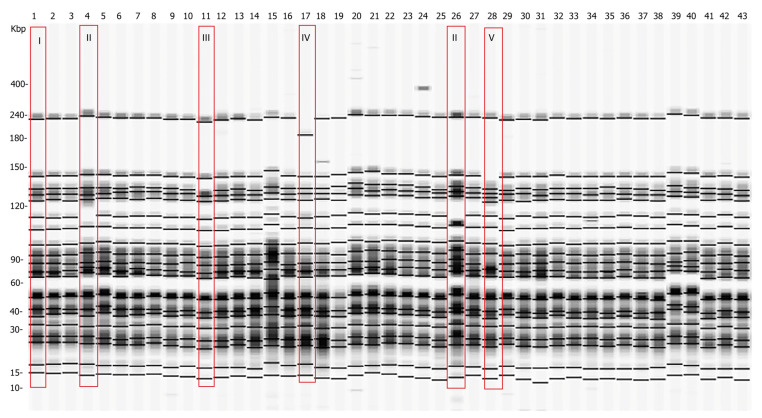
Pulsed-field gel electrophoresis pulsotypes of 43 *Y. enterocolitica* isolated strains after restriction with *Spe*I. Five pulsotypes were observed (surrounded by red rectangles). Pulsotypes II, III, IV, and V differ from the major pulsotype I, which is representative of all analyzed strains except strains 4, 11, 17, 26, and 28. The number of each lane corresponds to the number of the analyzed *Y. enterocolitica* strain (1–43). The pulse marker used was 50–1000 kb.

Forty-three *Y. enterocolitica* strains were examined by pulsed-field gel electrophoresis, using *SpeI* as a restriction enzyme. Five pulsotypes (I, II, II, IV, and V) with minor differences were detected as shown in Figure 2.

**Figure 3:**
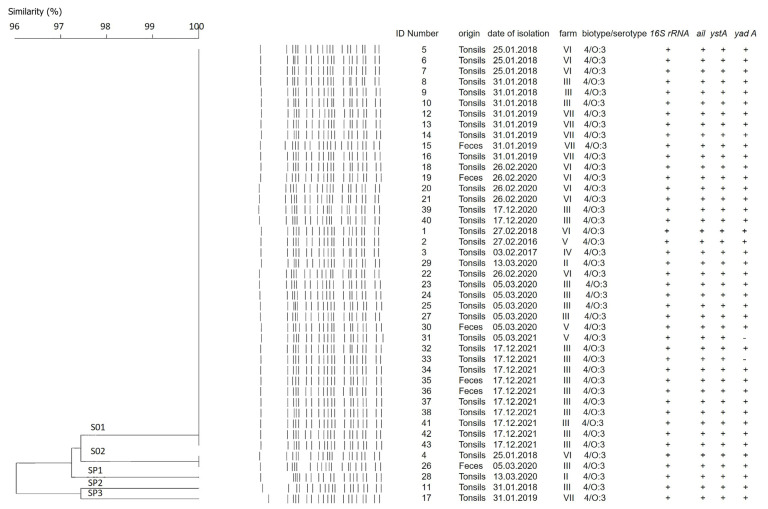
Dendrogram obtained derived by digesting *Y. enterocolitica* genomic DNA with *Spe*I. The DNA of 43 examined strains formed two different clusters, assigned S01 and S02, and three single pulsotypes, named SP1, SP2, and SP3. Clustering was calculated by the Unweighted pair group method with arithmetic mean (UPGMA) with 97% similarity. Dice correlation was with tolerance of 2.5% and optimization setting of 0.5. Additional information about dates of isolation, farm distribution, and virulence profile of the strains is given.

Additional clustering was performed at 97% similarity. The strains were organized in two clusters, labeled S01 and S02, and three single pulsotypes, SP1, SP2, and SP3 (Figure 3). The number of fragments within the pulsotypes obtained after restriction varied between 19 and 20, and the size ranged from 15 kb to 240 kb (Figure 2). As depicted in Figure 3, all strains proved to be closely related, sharing a high percentage of genetic similarity (over 92%). Cluster S01 included 38 (88.4%) of the 43 strains, being the predominant macro-restriction pulsotype (Figure 3). All *Y. enterocolitica* strains clustered within S01 were of serotype O:3 and were isolated from the tonsils and feces of pigs originating from the “positive” farms (Figure 1B, Farms II–VII). Two *Y. enterocolitica* strains with identical pulsotypes clustered in S02 (Figure 3). These originated from farms III and VI, which are located in different geographic regions (Figure 1B).

As shown in Figure 3, the strains isolated from tonsils and feces showed minor genomic differences. Clusters S01 and S02 were closely related to each other with 97.4% genetic similarity. One tonsil-derived strain, of a pig (originating) from farm II showed a single pulsotype SP1 (Figure 3). Pulsotype SP1 was closely related to S01 and S02 with 97.4% similarity. Single pulsotypes SP2 and SP3 were assigned to the remaining two *Y. enterocolitica* strains. Both strains originated from different farms and regions and were isolated from tonsils. Pulsotypes SP2 and SP3 were closely related with 97.4% similarity (Figure 3). Additionally, pulsotypes SP1 and SP2 were at equal distance from both S01 and S02 clusters (97.4% and 94.7%, respectively). SP3 was determined to be the most distant from other, and clustered as follows: to SP1 with 92.3% similarity, to SP2 with 97.4% similarity, to S01 cluster with 95% similarity, and to S02 cluster with 92.3% similarity (Figure 3).

### 2.5. Antimicrobial Susceptibility of Y. enterocolitica Strains

All 43 strains were tested for susceptibility to 15 antibiotics, belonged to 8 classes antibiotics and 1 unclassified antibiotic. The results are shown in Table 1. All strains were sensitive to ceftriaxone, amikacin, gentamicin, and ciprofloxacin. None of them was susceptible to ampicillin, novobiocin, cefamandole, and bacitracin. Forty-one strains were also sensitive to tetracycline, nalidixic acid, chloramphenicol, streptomycin, levofloxacin, trimethoprim/sulfamethoxazole, and doxycycline (Table 1). Two strains were observed to be multidrug resistant, demonstrating resistance to three other antibiotics: tetracycline, nalidixic acid, and chloramphenicol. One of them was also resistant to streptomycin and levofloxacin, and the other one to trimethoprim/sulfamethoxazole and doxycycline, respectively. In general, three resistance profiles were observed (Table 1). The most common profile was ampicillin/cefamandole/novobiocin/bacitracin resistance, detected in 95.3% of the strains. One strain (2.3%) demonstrated resistance to ampicillin/cefamandole/novobiocin/bacitracin/tetracycline/nalidixic acid/chloramphenicol/streptomycin/levofloxacin, and one strain (2.3%) was resistant to ampicillin/cefamandole/novobiocin/bacitracin/tetracycline/nalidixic acid/chloramphenicol/trimethoprim/sulfamethoxazole/doxycycline.

## 3. Discussion

The study revealed three main findings: (1) low prevalence of pathogenic *Y. enterocolitica* in healthy pigs from a slaughterhouse in Bulgaria with predominant *Y*. *enterocolitica* bioserotype 4/O:3; (2) high genetic similarity of the isolated *Y. enterocolitica* strains; and (3) three antimicrobial resistance profiles of the isolated *Y. enterocolitica* strains.

To the best of our knowledge, this is the first five-year study presenting data on the prevalence of *Y*. *enterocolitica* in pigs slaughtered in Bulgaria. The tested samples were collected in a single slaughterhouse, and originated from seven farms, located in four regions of Bulgaria. These data could be used for detailed epidemiological analysis of distribution patterns of pathogenic *Y*. *enterocolitica* in animals and humans in Bulgaria.

The estimated 6.7% prevalence of pathogenic *Y*. *enterocolitica* in healthy slaughtered pigs in this study is relatively lower compared to other European countries, such as Serbia (10.4%) [8], Italy (14% and 27.4%) [19,20], Belgium (23.5%) [9], Latvia and Lithuania (35%) [7], Croatia (43%) [17], Finland (60%) [21], Switzerland (85%) [12], and Spain (93%) [22]. The high prevalence in some countries could be explained by the different isolation methods, different age of the pigs, technical parameters, etc. It is well known that molecular methods are more sensitive and precise in comparison to culture methods [12,23]. Conventional microbiological methods of isolation with enrichment step followed by a PCR method for confirmation reduce the likelihood of false positive results due to dead cells [23,24]. Here, we combined microbiological, biochemical, and PCR methods with enrichment step in Peptone Sorbitol Bile Broth (PSB broth) to ensure the most successful possible isolation and detection of pathogenic *Y. enterocolitica* strains. To enhance recovery of pathogenic *Y. enterocolitica,* the enrichment period was reduced from five to two days, and isolation was performed on selective agar after alkali treatment of PSB broth [17,25]. Although we believe that the choice of farm type can also affect the detection of pathogenic *Y. enterocolitica,* specific types of farms that could be responsible for the occurrence of *Y. enterocolitica* among pigs were not explored herein and remain to be further researched. Porcine tonsils are well recognized as a source of pathogenic *Y*. *enterocolitica*, in view of their lymphoid tissue tropism. In line with previous studies [9,15,21,23], we primarily detected pathogenic *Y. enterocolitica* in pig tonsils, as well as in pig feces. Different studies have demonstrated that shedding of yersiniae in the feces increases in piglets younger than 30 days and decreases when pigs reach slaughter age [15,26].

*Y. enterocolitica* is a psychrophilic bacterium and can withstand cold temperatures over long periods of time [2]. Indeed, in the current study *Y. enterocolitica* strains were isolated during the cold period of the year similarly to other studies reporting isolation of *Y. enterocolitica* from tonsil samples mostly during cold months [27,28]. However, periodic recurrences of this pathogen (from March to August) cannot be excluded as it has been detected in pigs originated from the Saharan region [29] and from northern regions of Europe [21]. The latter author reported the highest prevalence of *Y. enterocolitica* in pig feces during July and August, assumed to be related to the higher consumption of pork, and an increased number of yersiniosis cases among humans during the warm period [21].

Sequencing data analysis revealed different similarity of our strains to the *16S rRNA* nucleotide sequence of *Y. enterocolitica* strain KNG22703, complete genome (GenBank accession number: CP011286.1), and the *16S rRNA* gene, partial sequence originated from uncultured bacteria clone 05-951_IBD.37307 (GenBank accession number: GQ965064.1). A very high similarity (98–99%) was established for most isolated strains in this study. Some of the isolated strains, such as *Y. enterocolitica* strains 18, 28, and 43, demonstrated similarities to KNG22703 of 97%, 93%, and 95%, respectively. Although small, the deviation of similarity among our isolates and the reference strain KNG22703 may be due to the different origin of the isolates, as KNG22703 has been adapted to humans.

Like other authors, we have shown that the most commonly isolated biotype among pigs is *Y. enterocolitica* biotype 4 [9,15,19,21,30], with the O:3 serotype confirmed in all 43 isolates. Moreover, in line with similar studies, we support the hypothesis of serotype O:3 predominance within biotype 4 of pig isolates. *Y. enterocolitica* 4/O:3 seems to be the most frequently detected and isolated bioserotype from healthy pigs in European counties [8,19,21], with the exception of the United Kingdom [27]. Our study identified *Y. enterocolitica* 4/O:3 as the only bioserotype isolated among healthy pigs in a slaughterhouse in Bulgaria. The prevalence of bio/serotype 4/O:3 poses a risk to human health, as this pathogen can easily enter the food chain through meat processing, spread to consumers, and eventually cause yersiniosis in humans [3]. Thus, our findings underline the importance of pigs in the epidemiology of yersiniosis.

The ail protein and yersiniabactin are important factors of virulence in *Y. enterocolitica*, both chromosomally encoded. Genes responsible for their expression—*ail* and *ystA,* respectively—are one of the most commonly used chromosomal targets for determination of pathogenicity. In our study, all genetically proven *Y. enterocolitica* strains harbored the genes of virulence *ail* and *ystA,* indicating a high pathogenic potential of the pig *Y. enterocolitica* isolates. Similarly, other studies identified *ystA* and *ail* genes with a high rate of presence among isolated *Y. enterocolitica* [8,19,20,30]. Of note, the presence of pYV is based on the detection of different genes, and the complete virulence of *Y. enterocolitica* depends on plasmid availability as well. The pYV is unstable and its detection is faced with some difficulties. However, many authors use *yadA* gene detection for plasmid confirmation [20,29]. We detected the plasmid-borne gene *yadA* by both PCR and culture methods, and, as expected, the PCR method revealed a higher detection rate.

It seems that *Y. enterocolitica* 4/O:3 is a less genetically diverse bioserotype. Indeed, we found a high degree of similarity between the macro-restriction pulsotypes of the *Y. enterocolitica* strains, suggesting a pronounced genetic homogeneity among the population of this species with only few isolates distinguishable from the predominant genotype. Our study found a 100% similarity within two clusters. This finding is in accordance with other studies which indicate a high degree of similarity and a minor genetic variation among 4/O:3 bioserotype strains, restriction enzyme applied (*Spe*I, *Not*I, or *Xba*I) [8,19,30]. In previous studies, we found that the genome of *Y. enterocolitica* is the most stable compared to the other two pathogenic species, *Y. pestis* and *Y. pseudotuberculosis* [31]. Studies on the genomic stability of *Y. enterocolitica* strains isolated from different countries of the world (belonging to bioserotype 4/O:3 and using the *Not*I restriction enzyme) revealed higher homogeneity of this bioserotype compared to serotypes O:5 and O:9 [32]. Obviously, the geographic location is an important factor for the spread of a given pulsotype and enables the emergence of new branches. The high genetic similarity observed in our study suggests well pronounced homogeneity and conservation in the genome structure of the strains. The majority of strains belong to close pulsotypes, pointing to the wide distribution of one genotype of *Y. enterocolitica* 4/O:3. The results showed the predominance of this genotype for all observed farms, and its persistence over time, indicating low genetic variation. The few other PFGE pulsotypes detected could be contamination with these genotypes on a farm level. Clustering with 100% similarity of *Y. enterocolitica* 4/O:3 isolated from palatine tonsils confirmed the relevance of palatine tonsils for direct *Y. enterocolitica* contamination in carcasses. [10]. The observed high similarity could be a disadvantage when tracking a possible outbreak because of the impossibility to establish a relationship between the isolated strain and the epidemiological outbreak [33]. Nevertheless, future studies should take into account circulating porcine *Y. enterocolitica* 4/O:3 genotypes, applying more discriminatory analyses.

The overuse of antibiotics in veterinary medicine as growth promoters in farm animals, including pigs, amplifies the significance of antimicrobial resistant *Y. enterocolitica.* Three profiles of resistance were detected among the isolated *Y. enterocolitica.* Our results showed that all tested strains were resistant to ampicillin, novobiocin, cefamandole, and bacitracin. Resistance to ampicillin is commonly reported for *Y. enterocolitica* strains isolated from pigs [8,10,15]. Since ampicillin belongs to the β-lactam penicillines, the resistance of *Y. enterocolitica* depends either on *β-*lactamase enzyme (BlaA and BlaB) production or on mutations of genes responsible for affinity to penicillin binding protein [34]. However, neither ampicillin, nor first-generation cephalosporin resistance of *Y. enterocolitica* O:3 is associated with plasmid presence [34], but probably with a mutation in the chromosome-coding genes, *blaA* and *blaB*, which deserves further investigation in detail. In 2 out of the 43 isolated *Y. enterocolitica* strains, we detected resistance also to tetracycline, nalidixic acid, and chloramphenicol, and only in 1 (out of 43) to streptomycin, trimethoprim/sulfamethoxazole, levofloxacin, and doxycycline. Unlike our data, resistance to sulfamethoxazole and streptomycin of *Y. enterocolitica* isolated from pigs and humans is continuously reported [10,17,20]. In line with our findings, resistance to chloramphenicol has not been frequently found [35], which contradicts other reports on the high rate of *Y. enterocolitica* resistance to chloramphenicol among slaughtered pigs in Northern Italy and Croatia [17,20,36], and among *Y. enterocolitica* clinical isolates that appeared during the time of the Swedish outbreaks [37]. There is some controversy regarding the resistance to tetracycline as well. Some authors reported none or a low proportion of isolates resistant to tetracycline [7,10,15,38], while others confirmed higher resistance to tetracycline of *Y. enterocolitica* strains [39]. A possible explanation could be associated with both chromosomal and plasmid-encoded mechanisms [37]. Nalidixic acid-resistant *Y. enterocolitica* has been frequently reported among pig isolates with resistance rates higher than the rates in our study, varying between 31% in Croatia, 49.1% in Northern Italy, and 62.5% in Malaysia [17,20,39]. It is known that the resistance to levofloxacin and to fluoroquinolones is rare [17] and could be reached by mutations of topoisomerase genes, which are targets of fluoroquinolones [40]. Overall, the resistance to quinolones is mediated by both chromosome- and plasmid-related mechanisms [16]. The resistance profiles against frequently used antibiotics found in our study are worrisome and should not be underestimated. Multi-resistant *Y. enterocolitica* strains can be a serious threat to human health after transmission in the food chain and food contamination.

## 4. Materials and Methods

### 4.1. Animals and Samples

The present study was conducted over a period of five years, spanning from January 2016 to December 2021. A total of 601 pigs were examined during the course of the sampling periods. Specifically, palatine tonsils *(veli palatini*) were collected from each of the 601 pigs. Both tonsils were aseptically removed immediately after evisceration using a sterile surgical blade. Additionally, a total of 189 fecal samples were collected from a subset of the slaughtered pigs (189 pigs only) and were aseptically harvested after colon incision. All samples were placed in sterile plastic bags, transported to the laboratory in a cooler bag at 4 °C and processed within 4 h after collection. Sampling was carried out during the slaughtering process in a single slaughterhouse located in Kostinbrod city (Sofia region), which serves pig farms across the country. The number of samples collected per visit in the slaughterhouse varied depending on the batch, with a target of collecting 50% of the total number of slaughtered pigs for the day. Importantly, all samples collected per day originated from a single farm. The study population comprised pigs from seven fattening pig farms (I, II, III, IV, V, VI, and VII) distributed across four different regions of Bulgaria, including Razgrad, Shumen, Stara Zagora, and Sofia Region. Specifically, farms I, II, and III were located in Razgrad, Shumen, and Stara Zagora, respectively, while farms IV, V, VI, and VII were located in the Sofia Region,. Sampling was conducted during two distinct periods: a cold period and a warm period. A total of 18 visits were conducted during the cold period and 8 visits during the warm period. The sampling period was chosen after taking into account the minimal and maximal monthly temperature of the cold period from October to March, and of the warm period from April to September.

### 4.2. Microbiological and Biochemical Tests for Detection of Yersinia enterocolitica

The presence of pathogenic *Y. enterocolitica* was detected according to ISO 10273:2003 [41] with some modifications, aiming to increase the detection efficacy of the test [25]. Briefly, tonsil tissue from each pig was aseptically cut into small pieces. A piece of each sample (tonsils and feces), approximately 12 g in weight from each, was suspended in peptone sorbitol bile salt (PSB) broth (Himedia, Mumbai, India) in a mass/volume ratio of 1:10. The suspension was homogenized for 4 min in a stomacher (Stomacher 80 Biomaster Lab, Seward, Worthing, UK). The enrichment period in PSB broth was reduced to 48 h at 28 °C. After alkali treatment with 4.5 mL 0.05% KOH solution and 0.5 mL PSB homogenate, for 20 s, a 10 µL aliquot of each sample was streaked onto *Yersinia* selective base agar (Oxoid, Hampshire, UK) supplemented with Cefsulodin-Irgasan-Novobiocin (CIN agar) (Oxoid, UK). The plates were incubated at 28 °C for 48 h. Small, smooth, flat colonies exhibiting a transparent appearance with a red central zone, commonly known as “bull’s eye” were selected for subculturing on Tryptic Soy agar (TSA, Difco, Atlanta, GA, USA) for further analysis. Preliminary identification included tests for urea hydrolysis and phenylalanine deamination on Christensen agar (Merck, Darmstadt, Germany) and Phenylalanine agar (Himedia, India), respectively, and tests for presence of catalase and cytochrome C oxidase. Briefly, urea positive, phenylalanine negative, catalase positive, and oxidase negative colonies were deemed presumptive *Y. enterocolitica*. Presumptive *Y. enterocolitica* were further identified biochemically by Microlatest ENTEROtest 24N (Erba Lacherna, Brno, Czech Republic), according to manufacturer’s instruction. The identification scheme included a test for: determination of arginine, lysine and ornithine decarboxylation, fermentation of carbohydrates, such as sucrose, lactose, trehalose, rhamnose, rafinose, melibiose, hydrolysis of citrate, production of hydrogen sulfide and utilization of manithol, arabitol, inositol. To confirm their identity, the colonies underwent *16S rRNA* gene identification.

### 4.3. DNA Isolation

Pure colonies of biochemically identified *Y. enterocolitica* were collected after overnight cultivation on TSA, and DNA extraction was completed using GenElute Bacterial Genomic DNA Kit (Sigma-Aldrich, St. Louis, MO, USA). Extracted DNA was quantified by a spectrophotometer (Quawell, Labgene Scientific SA, Châtel-Saint-Denis, Switzerland) and analyzed by gel electrophoresis. The DNA eluate in an appropriate amount was used as a template in PCR assays.

### 4.4. 16S rRNA Gene Identification

*The Yersinia 16S rRNA* gene was detected by genus-specific primers followed by DNA sequencing performed to all biochemically identified *Y. enterocolitica* isolates (n = 136). PCR was performed using Phire Green Hot start II PCR Master Mix (Thermo Fisher Scientific Baltics, Vilnius, Lithuania). The total reaction volume was 30 µL, containing 1.5 μL of each primer (with a final concentration of 0.5 µM), nuclease-free water (Thermo Fisher Scientific Baltics, Lithuania) and 25 ng/µL DNA. The amplifications were performed in a Thermal Cycler (Bio-Rad, Hercules, CA, USA), with details of the primer sequences, annealing temperature, and PCR conditions, as described in Table 2. DNA extracted from *Y. enterocolitica* 8081 (O:8) was used as a positive control, while master mix with HPLC water was used as a negative control. The PCR products (8 µL) were subjected to electrophoresis in a 1.5% agarose gel in 1% TBE and stained with PeqGreen (Peqlab Biotech, Hafenstr, Germany). For the positive control, DNA extracted from *Y. enterocolitica* 8081 (O:8) was used, and the negative control was a master mix with HPLC water. The positive PCR products for the *16S rRNA* gene were sequenced in both directions (Macrogene, Amsterdam, The Netherlands) and the sequenced data were compared with reference sequences of *Y. enterocolitica* in the database of the National Center for Biotechnology Information (NCBI) amplicons using Basic Local Alignment Search Tool (BLAST).

### 4.5. Biotyping and Serotyping Methods

*Yersinia enterocolitica* isolates confirmed by *16S rRNA* analysis were biotyped according to the biotyping scheme of ISO 10273:2003 using the reactions of: trehalose, tween esterase/lipase, pyrazinamidase, esculin/salicin, and indole [41]. Esculin/salicin hydrolysis and trehalose utilization were detected on Microlatest ENTEROtest 24N (Erba Lacherna, Czech Republic). Pyrazinamidase activity and tween esterase/lipase activity were assessed using pyrazinamidase agar (Himedia, India) and tween esterase test agar base (Himedia, India), supplemented with Tween 80 (Sigma Aldrich, Taufkirchen, Germany). Indole production was detected from trypthophan deamination using Kovacs’ Indole Reagent (Himedia, India). The serotype was determined by slide agglutination with the use of antisera for somatic antigens O:3 (Sifin, Berlin, Germany), O:5, O:8, and O:9 (BB NCIPD Ltd., Sofia, Bulgaria). Physiological saline was used as a negative control.

### 4.6. Phenotypic Test for Detection of Virulent Plasmid

The presence of pYV (plasmid *Yersinia* virulence) was studied by both absorptions of congo red in the Congo red-magnesium oxalate agar (CR-MOX) (Himedia, India) and autoagglutination on TSB (Difco, USA). CR-MOX agar plates were incubated at 28 °C and 37 °C for 24 h to 48 h and autoagglutination was performed at 28 °C and 37 °C as well.

### 4.7. PCR for Detection of Y. enterocolitica Virulence Genes

The genes encoding virulence determinants, such as the attachment-invasion locus (*ail),* and *Yersinia* heat-stable enterotoxin (*ystA*) and the plasmid-borne *Yersinia* adhesin (*yadA)* were analyzed by a PCR assay. Reactions were carried out using 2× PCR Buffer EURx Taq PCR Master Mix (containing 1.25 U Taq DNA polymerase, 1.5 mM MgCl_2_, 0.2 mM of each dNTPs, EURx, Poland), with a final concentration of 0.5 µM of each primer, 0.2 µg of DNA, and nuclease-free water. The primer sequences, annealing temperature, and PCR conditions are shown in Table 2. DNA extracted from *Y. enterocolitica* 8081 (O:8) pYV+ was used as a positive control and a master mix with HPLC water as a negative control.

### 4.8. Pulsed-Field Gel Electrophoresis

Macro-digestion of isolated pathogenic *Y. enterocolitica* strains (n = 43) was performed using the restriction enzyme *SpeI* (Sigma-Aldrich, Schnelldorf, Germany). PFGE was performed according to the PulseNet protocol for *Y. pestis* [42]. Bacteria grown for 24 h on TSB were centrifuged at 10,000× *g* for 10 min. Pellet was re-suspended in 2 mL Cell Suspension Buffer (100 mM Tris:100 mM EDTA, pH 8.0, Sigma-Aldrich, Schnelldorf, Germany) and bacterial concentration was adjusted by a spectrophotometer (Cole-Parmer, Vernon Hills, IL, USA) to optical density 1.3–1.4, measured at 610 nm wavelength. Agarose plugs were prepared with 200 µL bacterial suspensions, 10 µL proteinase K (0.5 mg/mL) (Sigma Aldrich, Germany), and 200 µL melted agarose (1% SeaKem Gold:1% SDS, Lonza, Bend, OR, USA and Thermo Fisher Scientific, Waltham, MA, USA, respectively). Cell lysis was performed with Cell Lysis/Proteinase K Buffer (50 mM Tris:50 mM EDTA, pH 8.0 + 1% Sarcosyl, Sigma Aldrich, Germany) and Proteinase K (final concentration of 0.1 mg/mL), followed by incubation at 54 °C for 2 h on a shaker water bath with constant and vigorous agitation (180 rpm). In order to inactivate proteinase K, the plugs were washed twice with sterile ultrapure water containing 1 mM Phenyl-methyl-sulphonyl-fluoride (PMSF) (Sigma-Aldrich, USA), followed by three washes in TE buffer (10 mM Tris-borate and 1 mM EDTA, pH 8.0). Washing steps were completed at 54 °C on a shaker bath. Plugs were subjected to individual restriction with *SpeI* (20 U/100 µL) for 18 h at 37 °C. Restriction fragments were separated by using Contour-clamped homogeneous electric field—Chef DR II system (Bio-Rad, USA) with 0.5× TBE buffer (45 mM Tris-borate and 1 mM EDTA, pH 8.3, Serva, Heidelberg, Germany) for 24 h at 16 °C, initial pulse of 2 s, final pulse 20 s with a voltage of 5 V/cm. Afterwards, the gels were stained with ethidium bromide (0.5 µg/mL) for 45 min, then de-stained in water for 1 h. Pulse marker (50–1000 kb DNA ladder, Sigma-Aldrich, USA) was applied as a molecular size standard. PFGE pulsotypes were analyzed using GelCompar software (Applied Maths, Keistraat, Brussels, Belgium). Clustering of strains was performed by unweighted pair-group method considering using arithmetic averages (UPGMA) with Dice bands correlation tolerance set on 2.5% and optimization of 0.5%.

### 4.9. Determination of the Antimicrobial Susceptibility of Y. enterocolitica Strains

Antimicrobial susceptibility tests were carried out by the standard disk diffusion method according to CLSI recommendations [18]. Standardized to 0.5 McFarland bacterial suspensions were plated on Muller-Hinton agar (Himedia, India) and commercial antimicrobial disks (BB-NCPID Ltd., Sofia, Bulgaria) were applied. Pathogenic *Y. enterocolitica* strains were tested for susceptibility to 15 antibiotics, belonging to different pharmacological classes (8 classes antibiotics and 1 unclassified antibiotic, according to [18]): tetracycline (30 µg), doxycycline (30 µg), amikacin (30 µg), gentamicin (10 µg), streptomycin (10 µg), ampicillin (10 µg), cefamandole (30 µg), ceftriaxone (30 µg), ciprofloxacin (5 µg), levofloxacin (5 µg), nalidixic acid (30 µg), chloramphenicol (30 µg), trimethoprim/sulfamethoxazole (1.25/23.75 µg), bacitracin (0.07 U), and novobiocin (5 µg). The diameter of Inhibition zone for each antibiotic was measured after incubation of the plates at 28 °C for 24 h. Results were interpreted as susceptible, intermediate, or resistant strains [43].

**Table 2:** Primers are used for PCR amplification.

Target Gene	Primers	Tm °C	Sequence (5′ to 3′)	Amplicon Length (bp)	Reference	PCR Conditions
*16S rRNA Yersinia enterocolitica*	YeI-6SrRNA YeII-6SrRNA	48 °C 45 °C	ATACCGCATAACGTCTTCG TTCTTCTGCGAGTAACGTC	328	[44]	95 °C for 5 min 35 cycles 94 °C for 30 s 47 °C for 30 s 72 °C for 1 min 72 °C for 10 min
*ail*	F-real 10A R-real 9A	55 °C 55 °C	ATGATAACTGGGGAGTAATAGGTTCG CCCAGTAATCCATAAAGGCTAACATAT	163	[45]	95 °C for 5 min 35 cycles 94 °C for 30 s 55 °C for 45 s 72 °C for 1 min 72 °C for 10 min
*ystA*	ystA-F ystA-R	63 °C 57 °C	AATGCTGTCTTCATTTGGAGC ATCCCAATCACTACTGACTTC	145	[29]	95 °C for 5 min 35 cycles 94 °C for 30 s 60 °C for 45 s 72 °C for 1 min 72 °C for 10 min
*yadA*	yadA-F yadA-R	63 °C 63 °C	CTTCAGATACTGGTGTCGCTGT ATGCCTGACTAGAGCGATATCC	849	[29]	95 °C for 5 min 35 cycles 94 °C for 30 s 63 °C for 45 s 72 °C for 1 min 72 °C for 10 min

## 5. Conclusions

Our results showed that 6.7% of the tested pigs slaughtered in Bulgaria were infected with pathogenic *Y. enterocolitica* strains with predominance of bioserotype 4/O:3. The isolated strains had high genetic similarity and most of them were sensitive to clinically important antibiotics. Our study indicated the significance of tonsils as predilection sites of pathogenic *Y. enterocolitica,* and the role of pigs as carriers of this zoonotic pathogen, suggesting the need of a surveillance system for monitoring *Y. enterocolitica* at the slaughterhouse level.

## Figures and Tables

**Table 1:** Antimicrobial susceptibility of *Y. enterocolitica* strains isolated from the slaughtered pigs. Three profiles of resistance to the selected antibiotics were observed.

Antibiotics		Resistance Profile		
Class *	Generic name	41/43	1/43	1/43
Penicillins	ampicillin	−	−	−
Cephems	cefamandole	−	−	−
	ceftriaxone	+	+	+
Aminoglycosides	amikacin	+	+	+
	gentamicin	+	+	+
	streptomycin	+	−	+
Phenicols	chloramphenicol	+	−	−
Tetracyclines	tetracycline	+	−	−
	doxycycline	+	+	−
Quinolones	nalidixic acid	+	−	−
	ciprofloxacin	+	+	+
	levofloxacin	+	−	−
Folate pathway antagonist	trimethoprim/ sulfamethoxazole	+	+	−
Aminocoumarins	novobiocin	−	−	−
Other	bacitracin	−	−	−

Legend: * Pharmacological classification defined according to Clinical and Laboratory Standards Institute (CLSI) [18]. (+) sensitive, (−) resistant.

## Data Availability

The data presented in this study are available on request from the corresponding author.
